# Annotations

**Published:** 1888-03-24

**Authors:** 


### Annotations.

**Bone-setting in the Black Country.**

Last summer a certain Mrs. Bagnall injured her wrist, and applied for treatment at the Walsall Hospital. The injury seems to have been a simple fracture, and the operating surgeon Sister Ellen. Mrs. Bagnall's fracture does not appear to have been a good case, for although the accident happened seven or eight months ago, the wrist is still too stiff to be used, and, so far as the evidence shows, is likely to remain so. That, of course, is a by no means uncommon result even in the hands of medical men. Nevertheless, it is clearly a circumstance to be avoided in so far as proper surgical skill may avail to avoid it. In reply to a question Sister Ellen stated that she should not send for the doctor for a broken wrist or arm, as she considered she was quite capable of setting either. She had set *upwards of a thousand arms, wrists, and legs* since she had been at the hospital. Dr. Maclachlan said both Sister Ellen and Sister Sophia "were trained in King's College. . . . Both the sisters could teach young students a good many things. . . . He was perfectly satisfied with the way the work was done. . . . He never heard of another hospital where the sisters did it." Thus far the evidence. There is nothing in the facts of which Sisters Ellen and Sophia need be ashamed. They both appear to be clever and capable women. It may be, too, that the circumstances at Walsall are peculiar; the honorary staff may be of necessity small; the funds may be so low as not to admit of the employment of one or more house surgeons. All these circumstances may be such as to justify to some extent the strange departure from wholesome and safe usage which has taken place, and apparently is to be continued. But we must point out that this settles the whole question of the justifiableness or otherwise of all kinds of unqualified practice. Here, with the full sanction of medical authority, is an unqualified person dealing with a thousand cases of broken limbs. If this kind of thing is to be done and approved when it suits the convenience of medical men, who will have the face to deny to patients and bone-setters their right to follow the same course when it suits their convenience? The real question at issue is this: Whether on the whole unqualified practice is good or bad for the public. If it be good, then let it be free everywhere and to all men. If it be bad, then let it be prevented everywhere, except when no other aid is available; and most of all, surely, should it be discountenanced in hospitals and by doctors themselves. The sisters, we repeat, are perhaps not to be blamed, but we think the medical men and the hospital managers at Walsall ought not to escape the severest censure.

**The "Lancet" and the College f Physicians.**

Under its new editorship the *Lancet* seems determined to show to the whole medical world what possibilities of foolishness lie open even to the most reputable newspapers. Emboldened by its success in inducing some of the more timid Fellows of th Royal College of Physicians to "dance to its piping," in a matter entirely unworthy of serious notice, a few months ago, it has now presumed to point out to that august body the man who of all others should in its judgment be elected to the post of president. That gentleman, whose just annoyance we do not wish to increase by unnecessarily dragging his name before the public, resents the interference of the *Lancet* with sober dignity. In a letter to that journal, he says: "You informed your readers in a leading article what you considered were my claims and qualifications for the honourable and distinguished post of President of the Royal College of Physicians. I ought, perhaps, to feel grateful for the very flattering terms in which those so-called claims were put forward; but when it is remembered that, by the regulations of the College, the choice of a President is to be made "*without any formal proposal or nomination*," I cannot but think that more respect would have been shown to the College, and more consideration for myself, if all reference to the subject had been avoided." The *Lancet* accepts the "rebuke" thus conveyed, but excuses itself on the plea that the Presidency of the College of Physicians is one of great dignity and importance, and that the selection of a president is not a "matter of interest to a few Fellows only, but one which the whole profession shares." That we readily admit, but the question is, Who are the fittest persons to nominate and appoint a president? Are the Fellows of the College, who periodically attend its meetings, and who in the course of years must gradually come to understand each other's characters well, and thus to know without any difficulty those of their number who are and those who are not fitted to maintain the dignity and authority which rightly appertain to the office of president; or are the editors of the *Lancet*, who merely know the Fellows of the College as any other decently-informed outsider knows them? What is the *locus standi* of the *Lancet* or any other newspaper on such a question as this? Is the paper striving in its old age to assume the dictatorship of the whole medical profession? Such an attempt would of course be as contemptible as it would be futile. But it not seldom happens that the founders of a successful enterprise leave their work in the hands of successors whose folly pulls down in a day what it has cost abler men the labour of years to build up. What kind of men do the editors of the *Lancet* hold the Fellows of the College of Physicians to be? They must surely consider them entirely destitute either of judgment or independence of mind if they suppose them capable of constantly taking their cue from a newspaper office. The Fellows of the College of Physicians are admittedly the flower of the medical profession. Will they condescend to the level of marionettes, and give a performance whenever and however the editors of a newspaper choose to pull the strings?

**Emigration and Self-help.**

It cannot be denied that the British Isles are too small to supply elbow-room to thirty millions of high-couraged and independent-minded Britons Were we a race of slaves like the "mild Hindoos," or the hapless negroes, the many might consent to work for the pleasure of the few, and when it was necessary, to starve for their pleasure also. Happily we are not yet, as a people, sunk to the level of a fatalistic despair. But the strongest man or race of men may be tamed, and nothing tends to extinguish the courage more quickly and more surely than the twin wolves of hunger and hopelessness. Those fierce creatures are now looking in upon us, and we must take steps for our own preservation. The Self-help Emigration Society is making manful efforts to put some of the homeless in the way of finding warm nests in secure trees in our own Colonies. The society has been in existence since 1885. In that year 150 emigrants were sent out at a cost of £850. In 1886 the sum of £1,447 was expended, and 360 were sent out. Last year showed still greater progress. The emigrants helped by the society numbered 545, and the money expended reached £2,713. Of this sum the emigrants themselves or their friends contributed £1,596, more than half. The society makes it a leading principle to help those who are willing to help themselves. It does not confine itself to money aid, which is sometimes the smallest difficulty the intending emigrant has to contend with. It meets him on the other side, and by its forty agents, all of whom are *honorary* helpers, and who are distributed over British North America from Vancouver to Quebec, puts him down finally in the place where he may remain, and helps him to the kind of work he is fittest for. The foreign land—which is indeed no foreign land, but a part of Greater Britain, where English laws and the English tongue prevail—is thus robbed entirely of its terrors. The emigrant leaves his home, such as it is, and a poor and prospectless career in this country, to find himself, after a long journey, established in a new and better home, with a prospect of unlimited prosperity if he be a man of brains and industry. The society asks for £5,000 during the present year, in order that it may largely extend its operations. There ought not to be the least difficulty in raising twice that sum. The rich should help freely, but still more should those come forward who have a little money left, but neither work nor prospect of any. They should promptly take time by the forelock and secure that better start which the possession of a little capital invariably gives.

